# MGDD: *Mycobacterium tuberculosis *Genome Divergence Database

**DOI:** 10.1186/1471-2164-9-373

**Published:** 2008-08-05

**Authors:** Anchal Vishnoi, Alok Srivastava, Rahul Roy, Alok Bhattacharya

**Affiliations:** 1 Center for Computational Biology and Bioinformatics, School of Information Technology, Jawaharlal Nehru University, New Delhi 110067, India; 2 Indian Statistical Institute, New Delhi 110016, India; 3 School of Life Sciences, Jawaharlal Nehru University, New Delhi 110067, India

## Abstract

**Background:**

Variation in genomes among different closely-related organisms can be linked to phenotypic differences. A number of mechanisms, such as replication error, repeat expansion and contraction, recombination and transposition can contribute to genomic differences. These processes lead to generation of SNPs, different types of repeat-based and transposons or IS-element-based polymorphisms, inversions and duplications and changes in synteny. A database of all the variations in a group of organisms is not only useful for understanding genotype-phenotype relationship but also in clinical applications. There is no database available at present that provides information about detailed genomic variations among different strains and species of *Mycobacterium tuberculosis *complex, organisms responsible for human diseases.

**Description:**

MGDD is a free web-based database that allows quick user friendly search to find different types of genomic variations among a group of fully sequenced organisms belonging to *M. tuberculosis *complex. The searches are based on data generated by pair wise comparison using a tool that has already been described. Different types of variations that can be searched are SNPs, indels, tandem repeats and divergent regions. The searches can be designed to find specific variations either in a given gene or any given location of the query genome with respect to any other genome currently available.

**Conclusion:**

Web-based database MGDD can help to find all the possible differences that exists between two strains or species of *M. tuberculosis *complex. The search tool is very user-friendly and can be used by anyone not familiar with computational methods and will be useful to both clinicians and researchers working on tuberculosis and other Mycobacterial diseases.

## Background

A large number of genomes of different strains and closely related species of pathogens have been sequenced and many others are in the process. A detailed analysis of these genomic sequences can help us to decipher and establish genotype to phenotype relationship. The organisms evolve through a series of molecular changes reflected in genomic sequences and some of these are evolutionarily selected based on survival in a specific ecological niche. Characterization of sequence alterations in closely related organisms can help us to understand genome evolution at the molecular level in short time span, for example emergence of new endemic strains in a few decades. Therefore it is important to catalog all the sequence differences between any two organisms so that these can be a basis for designing experiments linking phenotype to genotype. In pathogenic organisms such a database can be useful in identifying species and strain-specific markers that can be a basis for designing diagnostic reagents.

A number of molecular mechanisms have been described that are responsible for genomic changes [1]. These contribute to single nucleotide polymorphisms (SNP), variable number of tandem repeats, insertion/deletion with or without involving transposable elements and recombination. Many of these have been used as markers for identification of strains and diagnosis of pathogens [2-4]. *M. tuberculosis *is a major cause of morbidity and mortality throughout the world. Genomic variations in this organism have been used to type pathogenic strains in a limited scale [5,6]. A comprehensive database of all the genomic variations of *M. tuberculosis *is not currently available though some attempts have been made in this direction. For example, MTBreg (please see Availability & requirements for more information) covers variations that are detected using spoligotyping, MycoDB (please see Availability & requirements for more information) [7], MycoperonDB (please see Availability & requirements for more information) [8] and GenoMycDB (please see Availability & requirements for more information) [9] have some features that allow comparison between two genomes in a limited manner. In this report we describe a comprehensive database of genomic differences among strains and species of *Mycobacteria *belonging to the *M. tuberculosis *complex. The variations have been identified using ABWGC, a comparative genomic tool previously described by us [10]. We hope that this database will be useful to clinicians and basic scientists interested in understanding *Mycobacterial *diseases.

## Construction and content

The database contains pre-computed data derived from full genome sequences of *M. tuberculosis *strains H37Rv, CDC1551, H37Ra, F11, *Mycobacterium bovis *AF2122/97 and *M. bovis *BCG str. Pasteur 1173P2 using ABWGC [10]. The variations are categorized as SNPs, insertions, divergent regions (based on lack of sequence identity) and tandem repeats. All computations have been carried out in a pair-wise fashion. In some cases, such as SNPs the results differ depending upon the genome that has been used as a query in a pair of genomes. The database contains two sets of data pertaining to using each genome as query sequence. Insertion in one genome can be considered as a deletion in another genome, so the database contains only the insertions. If the insertions are due to known insertion elements and phage sequences these have been pointed out so that the information can be used for devising methods for better diagnosis and strain identification. Tandem repeats were identified using ABWGC and verified by "Tandem Repeat Finder" [11].

MGDD is implemented by using three- tier architecture. The web based application is created by using Apache web server which is connected to the database using MYSQL through an application layer written in Perl-CGI.

The information from MGDD can be obtained by selecting a specific query using the "search option" given in the MGDD browser.

## Utility and discussion

**Figure 1:**
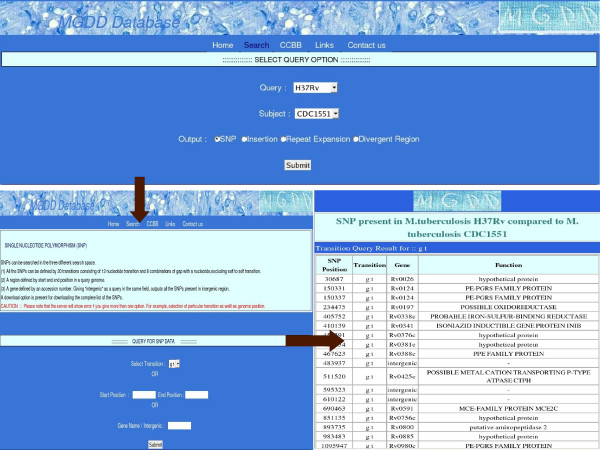
**A typical output of a query (SNP)**. The transition selected was 'gt' and *M. tuberculosis *H37Rv was compared with *M. tuberculosis *CDC1551.

MGDD has a web interface for the retrieval of genomic diversity information. A search can be initiated by first selecting genomes from the "Query" and "Subject" scroll down menu-bar. Currently there is information about six organisms and these can be selected in a pair-wise manner (Fig. 1). These organisms are:

*M. tuberculosis* CDC1551 (NC_002755.2)

*M. tuberculosis* F11 (NC_009565.1)

*M. tuberculosis* H37Ra (NC_009525.1)

*M. tuberculosis* strain H37Rv (NC_000962.2)

*M. bovis* AF2122/97 (NC_002945.3)

*M. bovis* BCG str Pasteur 1173P2 (NC_008769.1)

Each pair of organisms can be analyzed in two different ways by choosing each one as query and the other one as target genome. We recommend that a pair should be analyzed in both ways in order to get a comprehensive list of variations, particularly indels. After selection of organisms the type of variation needs to be selected from the search page. Currently there are four options available and one of these to be chosen among:

SNP, Insertion, Repeat expansion, Divergent regions

After submission of the selected information a detailed query page appears. For example, if SNP is selected the new page will ask for choosing one of the 20 different possible transitions in a user-defined menu-bar and the search can be made restrictive by specifying genomic coordinates or gene name (Fig. 1). The output would show all the indicated SNPs in the selected region along with annotation of genes that contain the SNPs (Fig. 1). For insertions, divergent regions and repeat expansion the query page has also the option of selecting output on the basis of size in nucleotides. There are four options at present and these are >10, 10–50, 50–100 and <100. Since one can select only one query at a time, an error message is displayed if more then one query is selected.

Table 1 gives the total data present in the database. However, the distribution of these changes among strains and species are different. For example, the number of SNPs between the two *M. tuberculosis *strains H37Ra and Rv are 588 and that between the two *M. bovis *strains are 1271 (Table 2). In general the number of variants, observed between the two *M. tuberculosis *strains were much less compared to that between the two *M. bovis *strains. This is consistent with the fact that *M. tuberculosis *strains H37Ra and Rv have been recently derived from H37 [12]. These differences are a result of evolutionary history of the organisms and can be useful to map all the potential mutation hotspots in these organisms.

**Table 1:** Statistics and data composition of MGDD

**Type of Data**	**Total number of entries.**
Divergence	829
Insertions	7865
Repeat expansion	578
SNP	68768

**Table 2:** Genomic variants in *M. tuberculosis *and *M. bovis *strains

	*M. tuberculosis *H37Rv compared to H37Ra	*M. bovis *compared to *M. bovis *strain BCG
SNP	582	1272
Divergent Region	1	4
Repeat expansion	3	12
Insertions	22	114

## Conclusion

In this report we describe MGDD, a database of genomic variants computed from fully sequenced organisms belonging to the *M. tuberculosis *complex. It contains data pertaining to SNP, insertions, repeat expansion and regions that show sequence divergence. Since MGDD is modular information regarding new genomes can be incorporated as and when the sequences become available. The search tool is simple and user friendly and allows one to locate a specific variation in any part of the genome or a gene.

## Availability and requirements

The web server can be accessed at .

MTBreg: 

MycoDB: 

MycoperonDB: 

GenoMycDB: 

## Authors' contributions

AV has implemented the ABWGC and identified the different divergent regions in two genomes and tested the database. AS has made the database and web based application. RR and AB helped in conceptualizing the database and writing the manuscript. All authors read and approved the final manuscript.
